# Emerging roles of growth factors in osteonecrosis of the femoral head

**DOI:** 10.3389/fgene.2022.1037190

**Published:** 2022-11-14

**Authors:** Zhenjia Che, Yang Song, Liwei Zhu, Tengyue Liu, Xudong Li, Lanfeng Huang

**Affiliations:** Department of Orthopaedics, The Second Hospital of Jilin University, Changchun, Jilin, China

**Keywords:** growth factor, osteonecrosis of the femoral head, gene polymorphism, tissue engineering, genetic engineering

## Abstract

Osteonecrosis of the femoral head (ONFH) is a potentially disabling orthopedic condition that requires total hip arthroplasty in most late-stage cases. However, mechanisms underlying the development of ONFH remain unknown, and the therapeutic strategies remain limited. Growth factors play a crucial role in different physiological processes, including cell proliferation, invasion, metabolism, apoptosis, and stem cell differentiation. Recent studies have reported that polymorphisms of growth factor-related genes are involved in the pathogenesis of ONFH. Tissue and genetic engineering are attractive strategies for treating early-stage ONFH. In this review, we summarized dysregulated growth factor-related genes and their role in the occurrence and development of ONFH. In addition, we discussed their potential clinical applications in tissue and genetic engineering for the treatment of ONFH.

## 1 Introduction

Osteonecrosis of the femoral head (ONFH) is a degenerative disease of the hip characterized by microfractures of the subchondral bone and subsequent collapse of the femoral head, resulting in hip dysfunction (Guerado and Caso, 2016; Liu et al., 2019). Because the femoral head is completely collapsed in 80% of untreated patients, ONFH has become one of the most severe challenges for orthopedic surgeons (Seamon et al., 2012). More than 20,000 new cases of ONFH are reported annually in the United States of America, and the prevalence of ONFH continues to increase (Hungerford, 2002; Mont et al., 2020). The incidence of ONFH is 1.4 per 100,000 population in the United Kingdom, which is similar to that reported in Japan (1.9 per 100,000 population) (Zhang Q. X. et al., 2021). In China, more than eight million individuals have been cumulatively diagnosed with ONFH (Zhao et al., 2020). Many factors are involved in the development of ONFH, including genetic factors, trauma, alcoholism, long-term or high-dose treatment with glucocorticoids, long-term diving, sickle cell disease, and other environmental factors (Mont et al., 2015; Wei et al., 2019). However, the exact mechanism underlying the development of ONFH remains largely unknown. With this regard, scholars have proposed various theories, including intravascular coagulation (Wang et al., 2010; Kumar et al., 2014), disorders of lipid metabolism (Zeng et al., 2017), increased intraosseous pressure (Mukisi et al., 2009), osteocyte apoptosis (Jilka et al., 2013), genetic polymorphisms (Zheng et al., 2014), and immune factors (Tian et al., 2014). Therefore, it is important to understand the pathogenesis of ONFH for its prevention, diagnosis, and effective treatment. Clinically, the treatment of early-stage ONFH is mainly restricted to physical interventions, pharmacotherapy, surgical core decompression (CD), porous tantalum rod implantation, osteotomy, and vascularized bone grafting (Zhao et al., 2020). However, the results of these hip preservation techniques are unsatisfactory (Sadile et al., 2016). After collapse, total hip arthroplasty (THA) remains the only treatment strategy for relieving pain and restoring joint functionality (Migliorini et al., 2021). Although surgical methods and biomaterials have been developed for THA, the expensive treatment cost, huge surgical trauma, serious complications such as periprosthetic infection and aseptic loosening, and limited survival of the prosthesis remain serious challenges (Pollock et al., 2016; Molloy et al., 2017; Swarup et al., 2018). Given these difficulties, it is essential to develop new treatment modalities to delay or reverse ONFH.

Vascular endothelial growth factor (VEGF), bone morphogenetic proteins (BMPs), transforming growth factor (TGF), insulin-like growth factor (IGF), hepatocyte growth factor (HGF), platelet-derived growth factor (PDGF), and fibroblast growth factor (FGF) are growth factors that play a significant role in regulating cell differentiation, apoptosis, morphogenesis, embryogenesis, angiogenesis, wound healing, hematopoiesis, inflammation and infection, tumorigenesis, and immunity in humans (Kempen et al., 2010; Goel and Mercurio, 2013; Rijcken et al., 2014; Mitchell et al., 2016). Increasing evidence suggests that growth factors can regulate bone development and regeneration and are directly involved in the pathogenesis of numerous orthopedic conditions, such as intervertebral disc degeneration, osteoarthritis, osteoporosis, and osteosarcoma (Zhang et al., 2017; Huang et al., 2018; Morris and Edwards, 2021; Zhong et al., 2021). Several recent studies have suggested that growth factors are involved in the development of ONFH. In addition, the pathological condition of ONFH is characterized by fibrosis and inflammation, which are associated with several growth factors that affect osteoblast activity (Zhu et al., 2017). Scholars have developed novel therapeutic modalities for ONFH based on the unique advantages of growth factors.

In this review, we summarized dysregulated growth factor-related genes and their pathogenic role in ONFH and discussed the potential applications of growth factors or related genes for the treatment of ONFH.

## 2 Association between the polymorphisms of growth factor-related genes and osteonecrosis of the femoral head

Studies have elucidated the genetic factors of ONFH, suggesting an alternative hypothesis to the development of the disease. Several ONFH susceptibility genes have been discovered in multiple populations. In this section, the association between the polymorphisms of growth factor-related genes and ONFH is summarized in Table 1.

**TABLE 1:** The association of the growth factor related genes polymorphisms with ONFH.

Growth factor-related gene	SNP	Risk association	Patient population	Reference
VEGF	−634G/C	Risk	Korean population 317 patients, 497 healthy individuals	Kim et al. (2008)
	−634G/C	Risk	Chinese population 220 patients, 220 healthy individuals	Liu et al. (2012)
	−634C/G	Risk	Chinese population 489 patients, 1,273 healthy individuals	Ma et al. (2018)
IGF-1	rs35767	Protective	Chinese population 101 patients, 128 healthy individuals	Wang et al. (2019)
	rs5742714	Risk		
	rs972936	Risk		
	c.15+3G>A	Risk	Two first-degree relatives with ONFH	Xu et al. (2021)
IGFBP-3	rs2453839	Risk	Korean population 60 patients, 300 healthy individuals	Hong et al. (2010)
	rs2453839	Risk	Chinese population 49 patients, 42 healthy individuals	Song et al. (2012)
	rs3110697	Risk	Chinese population 182 patients, 179 healthy individuals	Song et al. (2016)
	rs2453839	Risk		
	rs2132572	Risk	Chinese population 200 patients, 177 healthy individuals	Song et al. (2017)

### 2.1 Vascular endothelial growth factor

VEGF is a major angiogenic factor and prime regulator of endothelial cells, which regulates endothelial cell proliferation, maintains endothelial cell function, and promotes vascular regeneration (Atis et al., 2012). The human VEGF gene is located on chromosome 6p21.3 and is alternatively spliced by eight exons to form a family of proteins. It has more than 30 single-nucleotide polymorphisms (SNPs), with 10 polymorphisms in its promoter region; among which, polymorphisms at the VEGF −634G/C, +936C/T, and −2578C/A have been shown to alter plasma VEGF levels (Andraweera et al., 2013; Chedraui et al., 2013). In addition, these polymorphisms can influence the etiology of various pathological conditions such as diabetic retinopathy (Awata et al., 2002; Yang et al., 2020), prostate cancer (McCarron et al., 2002; Yang et al., 2020), and breast cancer (Krippl et al., 2003).

Insufficient blood supply to the femoral head has been suggested as a pathogenic mechanism of ONFH (Zhao K. et al., 2021). Several studies have investigated the relationship between polymorphisms in the VEGF gene and the risk of ONFH; however, the results are inconsistent and contradictory. Kim et al. (2008) focused on the 5′-untranslated region (UTR), a promoter region, and the 3′-UTR of VEGF for genetic analysis of ONFH. They examined the genotypes and allele frequencies of three SNPs (−2578C>A, −634G>C, and +936C>T) in the VEGF gene in 317 patients with ONFH and 497 control individuals in a Korean population. They identified a significant association between the −634G>C polymorphism and the risk of ONFH; however, the genotypes and allele frequencies of +2578C>A and −936C>T polymorphisms between the two groups of patients were not significantly different. Further stratified analysis based on sex showed that the −634G>C genotype was significantly associated with a high risk of ONFH among male patients. In addition, the allele frequency of the C allele of −634G>C in female patients was similar to that in male patients; however, the allele frequency was not significantly different between female and control patients. This result can be attributed to the small number of female patients in the study. Liu et al. (2012) evaluated the association between the VEGF −634G/C polymorphism and ONFH in 220 unrelated patients with nontraumatic ONFH and 220 unrelated control individuals in a Chinese population. Their results suggested that the VEGF −634G/C CC genotype is a risk factor for ONFH.

Previous studies have only focused on the relationship between polymorphisms in the VEGF gene and nontraumatic ONFH and have not addressed the specific etiology. Lee et al. (2012) investigated the association between steroid-induced ONFH and functional VEGF gene polymorphisms (−2578A/C, −1154A/G, −634C/G, and −405C/G) in 160 patients (86 idiopathic ONFH and 74 steroid-induced ONFH) and 160 sex- and age-matched control individuals in a Korean population. Low-inducing VEGF haplotypes (C-G-G-C and A-G-G-C) conferred increased risk, whereas high-inducing haplotypes (C-G-C-G and A-A-G-G) had a protective effect on the development of steroid-induced ONFH. In the study, patients with ethanol-induced ONFH were excluded because chronic ethanol exposure can increase VEGF expression. Ma et al. (2018) analyzed 22 SNPs in VEGF in 1,762 Chinese individuals (489 patients with ONFH and 1,273 control individuals) and found that the −634C/G SNP was significantly associated with alcohol-induced ONFH but not with steroid-induced ONFH. In addition, the −634C/G SNP was found to be associated with the disease status of ONFH. Some SNPs, such as −2578A/C and −1154A/G, which have been shown to be significantly associated with ONFH in other studies, were found to be only surrogates of −634C/G.

### 2.2 Insulin-like growth factor-1

IGF-1, a polypeptide containing 70 amino acid residues, is found in almost all tissues in mammals and can accelerate cell proliferation and differentiation, functioning as a mitosis promoter for many types of cells (including osteoblasts) (Liu et al., 2018). Several studies have shown that IGF-1 polymorphism is associated with various human diseases, such as acne severity (Rahaman et al., 2016), diabetes (Wang et al., 2022), and gastric cancer (Meisami and Jalilvand, 2020). In addition, IGF-1 can affect bone tissues *via* several pathways, mainly involving osteogenesis and bone metabolism (Gao et al., 2015). Dysregulation of IGF-1 may lead to osteoblast aging and metabolic bone diseases (McMichael et al., 2017). In an animal study, IGF-1-knockout mice had smaller cytoskeletons and fewer cells than wild-type mice. In addition, the osteoblasts of IGF-1-knockout mice were more predisposed to apoptosis, and their osteogenic ability was significantly weakened (Sabokbar et al., 2016). Furthermore, IGF-1 expression is closely related to tissue repair in ONFH (Chen et al., 2008). In a study on rabbits with steroid-induced ONFH, IGF-1 was measured *via* enzyme-linked immunosorbent assay at 4, 8, and 16 weeks, and the results revealed that IGF-1 expression began to increase 4 weeks earlier than the appearance of abnormal bone marrow tissue in rabbits (Saygun et al., 2012; Xu et al., 2021).

Wang et al. (2019) investigated the genetic association between the IGF-1 polymorphisms rs35767, rs5742714, and rs972936 and susceptibility to ONFH among the Han Chinese population (101 patients with ONFH and 128 healthy individuals). Significant differences were observed in the three polymorphisms between ONFH and control groups. The results suggested that the IGF-1 polymorphisms rs35767 and rs5742417 play a protective role in ONFH susceptibility, whereas the polymorphism rs972936 enhances the risk of ONFH among the Han Chinese population.

A few previous studies have reported on familial ONFH, and most of them focused on the gene locus of COL2A1 (Kishiya et al., 2014). Xu et al. (2021) reported two first-degree relatives with ONFH and analyzed ONFH-related genes *via* whole exome sequencing (WES). They found a heterozygous mutation (c.15+3G>A) in IGF1 in the family, resulting in incorrect site recognition and abnormal mRNA splicing of the gene, leading to an abnormal quantity or structure of amino acids. Therefore, mutant IGF1 may be the disease-causing gene in the family. In addition, this finding reveals that IGF-1 is a potential marker for the pathogenesis and molecular diagnosis of ONFH.

### 2.3 Insulin-like growth factor binding protein 3

The insulin-like growth factor binding protein 3 (IGFBP-3) gene is located on chromosome 7p12.3 and is a member of the IGFBP family. It is a significant carrier of serum IGF-1 and inhibits IGF-1 activity by competitively binding to ligands (Yamada et al., 2010). IGFBP-3 forms a ternary complex with IGF acid-labile subunit and either IGF-1 or IGF-2. In this form, it circulates in plasma, prolonging the half-life of IGFs and altering their interaction with cell surface receptors (Ceda, 1995; Dar et al., 2010; Moya-Angeler et al., 2015).

IGFBP-3 polymorphisms are correlated with tumor risk but are rarely involved in orthopedic diseases. A recent study showed that an adenovirus vector containing IGFBP-3 complementary DNA inhibited the activity of nuclear factor kappa B (NF-κB), production of chemokines, and secretion of matrix metalloproteinases in cultured fibroblast-like synovial cells and a mouse model of collagen-induced arthritis (CIA). In addition, the vector decreased the severity of arthritis and pathological changes in mice, suggesting that IGFBP-3 may reduce inflammatory bone lesions (Lee et al., 2014).

In 2010, Hong et al. (2010) first reported that the polymorphism rs2453839 in the IGFBP-3 gene and high IGFBP-3 levels in serum were closely associated with the risk of ONFH in a Korean population. Subsequently, in 2012, Song et al. (2012) validated that the genotypes of IGFBP-3 rs2453839 correlated with the increased risk of bilateral hip lesions in 49 patients with ONFH and 42 healthy individuals in a Chinese population. In 2016, Song et al. (2016) further demonstrated that the genotypes of both rs3110697 and rs2453839 were associated with a higher risk of ONFH and the clinical stage of ONFH in a case-control study involving 361 patients (the study did not include any patient from their previous study). Initially, they analyzed the association between ONFH development and the genotypes, allele frequencies, and haplotypes of rs2453839 and rs3110697. The results showed that the recessive model of rs3110697 and the dominant model of rs2453839 were significantly associated with an increased risk of ONFH. Furthermore, the correlation between IGFBP-3 polymorphisms and the clinical phenotypes of ONFH was analyzed, which revealed that the CT genotype of rs2453839 is a risk factor and the CC and TT genotypes of rs2453839 are protective factors for the progression of hip lesions in ONFH. In addition, the serum protein expression of IGFBP-3 and IGF-1 was closely related to IGFBP3 function, and both serum IGFBP-3 and IGF-1 levels were significantly higher in the ONFH group than in the control group. Serum IGF-1 levels were significantly lower in patients with bilateral hip than in patients with unilateral hip lesions, suggesting the possible role of IGF-1 in the progression of hip lesions in ONFH. In the following year, Song et al. (2017) further revealed the association between the genotype of IGFBP-3 rs2132572 and the risk of ONFH in a case-control study involving 370 patients.

## 3 Association between growth factor-related signaling pathways and osteonecrosis of the femoral head

### 3.1 TGF-β signaling pathway

The TGF-β superfamily comprises a large group of growth factors, such as TGF-β, activins, inhibins, growth and differentiation factors (GDFs), and BMPs (Peng, 2003). Among these factors, TGF-β is an essential cytokine involved in the function and metabolism of osteoblasts, which can promote osteoblast mitosis, reduce collagen loss, increase the rate of bone deposition, and promote osteoblast differentiation (Wu et al., 2016). In addition, TGF-β signaling is involved in most cellular processes, especially in the early proliferation, differentiation, maturation, and apoptosis of osteoblasts (Chen et al., 2012). At present, TGF-β is a major focus of research on osteogenesis-related signaling pathways (Sun et al., 2018). Smad proteins are intracellular kinase substrates of the TGF-β receptor and are responsible for the signal transduction of BMPs and TGF-β during osteogenesis and chondrogenic differentiation (Zhang, 2018). TGF-β signaling regulates osteoclast development and osteoblast differentiation in ONFH (Rahman et al., 2015). Experimental studies have validated the association between the TGF-β/Smad signaling pathway and ONFH. Li et al. (2020) reported a significant decrease in TGF-β1 expression in femoral head specimens collected from adult patients with nontraumatic ONFH, which indicates the involvement of the abnormal TGF-β/Smad pathway in the pathological process of ONFH. Furthermore, TGF-β-related signaling pathways may serve as therapeutic targets for nontraumatic ONFH (Tao et al., 2017). Studies have shown that regulation of the TGF-β signaling pathway by non-coding RNAs contributes to the development of ONFH. Tian et al. (2020) examined the femoral head tissues of 33 patients with steroid-induced ONFH and 33 patients with femoral neck fracture *via* immunohistochemical analysis, RT-PCR, and western blot. The results revealed that miR-141 expression was high and TGF-β2 expression was low in the femoral head tissues of patients with ONFH, and TGF-β2 was identified as a direct target of miR-141. In addition, a rat model of ONFH was constructed by injecting hormones, and the relationship between miR-141 and TGF-β2 in ONFH was further validated through animal experiments. Decreased expression of miR-141 or overexpression of TGF-β2 inhibited apoptosis in bone cells of rats with ONFH; increased the expression of osteoprotegerin (OPG), B-cell lymphoma 2 (Bcl-2), BMP-2, and runt-related transcription factor 2 (Runx2); and decreased the expression of osteoprotegerin ligand (OPGL), Bcl-2-associated X (Bax), and receptor activator of NF-κB (RANK) in the femoral head tissues of rats with ONFH.

The Smad7 protein acts as an intracellular inhibitory protein that antagonizes signal transduction among the TGF-β family members (Lukas et al., 2017). Fang et al. (2019) demonstrated that miR-15b expression was low in bone marrow-derived mesenchymal stem cells (BMSCs) of patients with ONFH, which significantly upregulated the protein expression of Smad7 and inhibited the TGF-β signaling pathway, eventually weakening the osteogenic differentiation ability of BMSCs. In addition, Bai et al. suggested that miR-27a regulates steroid-induced ONFH *via* TGF-β/Smad7 signaling (Bai et al., 2019). Therefore, aberrant regulation of the TGF-β/Smad7 signaling pathway may be a potential mechanism underlying the increased risk of ONFH (Hao et al., 2021).

Regulation of TGF-β-related signaling pathways can effectively inhibit osteoblast apoptosis, accelerate osteogenic differentiation, and promote bone repair and regeneration in ONFH. The abovementioned studies offer a theoretical basis for further investigation of the pathogenesis, etiology, and treatment of ONFH.

### 3.2 Bone morphogenetic protein signaling pathway

BMPs belong to the TGF-β superfamily, a group of highly conserved homologous signaling proteins that are involved in embryogenesis, organogenesis, cell proliferation, and stem cell differentiation (Li et al., 2016). To date, approximately 20 BMPs have been identified and characterized, including various isoforms from BMP2 to BMP16, which regulate bone formation and development (Tian et al., 2015). BMP signaling is transduced through type I (BMPRI) and type II (BMPRII) receptors, which interact to form a functional complex to initiate further signaling pathways (Koenig et al., 1994). On the one hand, activated BMPRI phosphorylates Smad-dependent signaling pathways (Bao et al., 2015), and regulation of the BMP-2/Smad/Runx2 pathway increases or decreases bone mass during bone tissue growth. In addition, the BMP-2/Smad/Runx2 pathway participates in bone formation and reconstruction, osteogenic differentiation of stem cells, maturation of osteoblasts, and secretion and mineralization of the extracellular matrix (Cui et al., 2017). On the other hand, BMP receptors activate non-Smad-dependent signaling pathways, namely, the p38 mitogen-activated protein kinase (MAPK), extracellular signal-regulated kinase (ERK), and c-Jun N-terminal kinase (JNK) signaling pathways (Guicheux et al., 2003). Subsequently, BMP signaling stimulates the expression of the main osteogenic transcription factors Runx2, distal-less homeobox 5 (Dlx5), and osterix (Osx) (Lee et al., 2003). Therefore, the BMP signaling pathway plays a critical role in inducing osteogenesis.

Studies have demonstrated that miR-23a-3p is the most significantly upregulated miRNA in patients with ONFH, which is significantly downregulated during osteogenic differentiation (Dong et al., 2017). Overexpression of miR-23a-3p inhibits and its downregulation enhances the osteogenic differentiation of BMSCs (Dai et al., 2019). Consistent with previous studies, Zhang X. Y. et al. (2021) reported that miR-23a-3p expression was significantly increased in rat models of ONFH. In addition, miR-23a knockdown promoted the viability and osteogenic differentiation of BMSCs and increased the mRNA and protein expression of BMP-2, BMP-4, Runx2, Smad5, Wnt1, and β-catenin in BMSCs and rat models of ONFH. Changes in the expression of these factors regulated the BMP-2/Smad5/Runx2 and Wnt/β-catenin pathways.

In addition to the suppression of osteogenic differentiation, endothelial dysfunction may significantly contribute to the progression of ONFH. Huang et al. (2022) reported that the protein expression of BMP-2/6/7 and Smad-1/5/8 was decreased in femoral head tissues with glucocorticoid (GC)-induced osteonecrosis and GC-stimulated bone microvascular endothelial cells (BMECs). However, silencing of GREM2, a specific antagonist of BMP-2, reversed the suppressive effects of GC on BMP-2/6/7 and Smad-1/5/8. Therefore, the BMP signaling pathway is involved in the development of ONFH and can be targeted to prevent the progression of ONFH.

## 4 Therapeutic applications of growth factors for osteonecrosis of the femoral head

**SCHEME 1:**
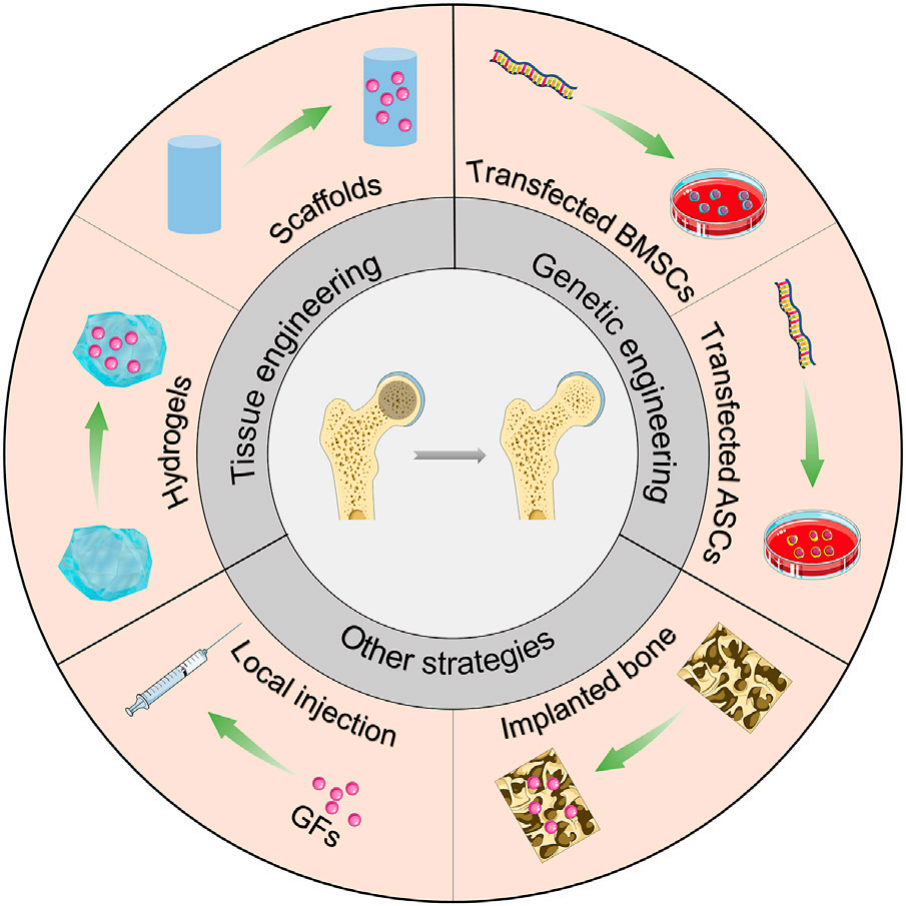
Therapeutic application of growth factors for ONFH.

Osteogenic and angiogenic-related growth factors are promising tools for treating hip preservation in ONFH. In this section, we summarize the potential applications of growth factors or related genes for the treatment of ONFH (Scheme 1).

### 4.1 Tissue engineering

To improve the regeneration of bone tissue in conventional treatment, growth factors can be combined with bone tissue engineering materials during surgery to achieve effective bone regeneration and osteoinduction. The combination of different growth factors and tissue engineering materials for the treatment of ONFH has been summarized in Table 2.

**TABLE 2:** The different growth factors combined with genetic engineering strategies for treating ONFH.

Therapeutic strategy	Growth factor	Animal	Associated cells	Delivery strategy	Regeneration results	Reference
Tissue engineering	VEGF	Rabbits	BMSCs	LiCPP/GM/VEGF scaffold + CD	Improved osteogenesis and angiogenesis Contributed to bone repair in GIONFH	Luo et al. (2019)
	BMP-2 + VEGF	Rabbits	BMSCs	BMP/VEGF/PLGA/CPC scaffold + CD	Enhanced bone reconstruction and blood vessel regeneration	Zhang H. X. et al. (2016)
	BMP-2	Pigs	BMSCs	BMP-2 + RADA16 hydrogel	Controlled the dissemination of biologically active BMP-2 Stimulated the growth of BMSCs	Phipps et al. (2016)
	rhFGF-2	Rabbits	—	rhFGF-2 + gelatin hydrogel	Increased new bone formation and prevented the femoral head from collapsing Increased the Harris hip score Reduced the pain level	Kuroda et al. (2010) and Kuroda et al. (2016)
Genetic engineering	BMP-2	Goats	BMSCs	BMP-2-transduced BMSCs + β-TCP + CD	Increased the volume of new bone Improved mechanical properties	Tang et al. (2007)
	BMP-2	Rabbits	BMSCs	BMP-2-transduced BMSCs + magnesium alloy rods	Recovery with normal running ability	Katiella et al. (2016)
	VEGF-165	Dogs	BMSCs	VEGF-165-transduced BMSCs + CD	Increased new bone formation and neovascularization	Hang et al. (2012)
	HGF	Rabbits	BMSCs	HGF-transduced BMSCs + CD	Enhanced blood vessel regeneration and bone reconstruction Recovery with decreased empty lacunae and hematopoietic tissue	Wen et al. (2008) and Wen et al. (2012b)
	FGF-2	Rabbits	BMSCs	FGF-2-transduced BMCSs/XACB + CD	Improved OPG expression Inhibited TNF-α expression Promoted angiogenesis and bone formation	Peng et al. (2018), Zhang et al. (2018) and Peng et al. (2019)
	PDGF-BB	Rabbits	BMSCs	PDGF-BB-transduced BMCSs + CD	Reduced the progression of osteonecrosis Enhanced bone regeneration and angiogenesis	Guzman et al. (2021)
	BMP-2 + VEGF	Rabbits	BMSCs	VEFG/BMP-2-transduced BMSCs + CD	Elevated the number and quality of new bones Accelerated bone repair	Ma et al. (2015)
	BMP-2 + FGF	Dogs	BMSCs	BMP-2/FGF-transduced BMSCs + DBM	Improved neovascularization density Increased compressive and bending strength	Peng and Wang (2017)
	BMP + VEGF	Rats	ASCs	BMP-2/VEGF-transduced ASCs	The optimal ratio of BMP-2-to-VEGF for enhancing both osteogenesis and angiogenesis was 9:1	Lee et al. (2019)
Other strategies	rhBMP-2	—	—	rhBMP-2 + light bulb procedure	Improved the clinical efficacy and quality of bone repair	Sun et al. (2014) and Shi et al. (2017)
	VEGF	Dogs	—	Single injection and osmotic micropump	Enhanced bone tissue remodeling and new bone formation	Dailiana et al. (2018)
	BMP-2	Pigs	—	BMP-2 + IB	Decreased femoral head deformity Stimulated bone formation	Vandermeer et al. (2011) and Aruwajoye et al. (2017)
	BMP-2	Rats	MC3T3-E1	BMP-2/nanofiber scaffold + LIPUS	Improved load-carrying capacity, bone formation, angiogenesis, and differentiation	Zhu et al. (2020)

#### 4.1.1 Scaffolds

CD and bone grafting are effective measures for the clinical treatment of pre-collapsed ONFH (Brown et al., 1993; Kang et al., 2007; Chen et al., 2009). However, therapeutic outcomes are usually unsatisfactory because the functions of osteoblasts, osteocytes, and osteoclasts are impaired and the adipogenic potential of BMSCs is upregulated during the development of ONFH (Sheng et al., 2007; Xie et al., 2011). Bone tissue engineering has made rapid progress in recent years and may offer new therapeutic avenues for ONFH. Bone repair and vascular regeneration in ONFH are associated with various growth factors such as BMP and VEGF (Street et al., 2002; Kerachian et al., 2006; Yu et al., 2010; Garcia et al., 2012). Therefore, scholars are using the combination of bone tissue-engineered scaffolds and growth factors to promote bone repair and angiogenesis, thereby alleviating or reversing ONFH. Luo et al. (2019) synthesized a novel calcium polyphosphate (CPP) composite scaffold containing Li and VEGF-loaded gelatin microspheres (LiCPP/GMs/VEGF). The porous LiCPP/GMs/VEGF scaffold had good mechanical properties that met the strength requirements of cancellous bone (2–12 MPa). The scaffold continuously released Li^+^ and VEGF, showing favorable cell biological activity. When the scaffold was added to BMSC cultures, it significantly increased cell proliferation, osteogenesis, and angiogenesis. Furthermore, the scaffold stimulated the expression of osteogenic and angiogenic factors to alleviate ONFH *in vivo* in a rabbit model of ONFH. These results suggest that the LiCPP/GMs/VEGF scaffold improves the efficacy of CD and has potential value for the treatment of ONFH.

**FIGURE 1:**
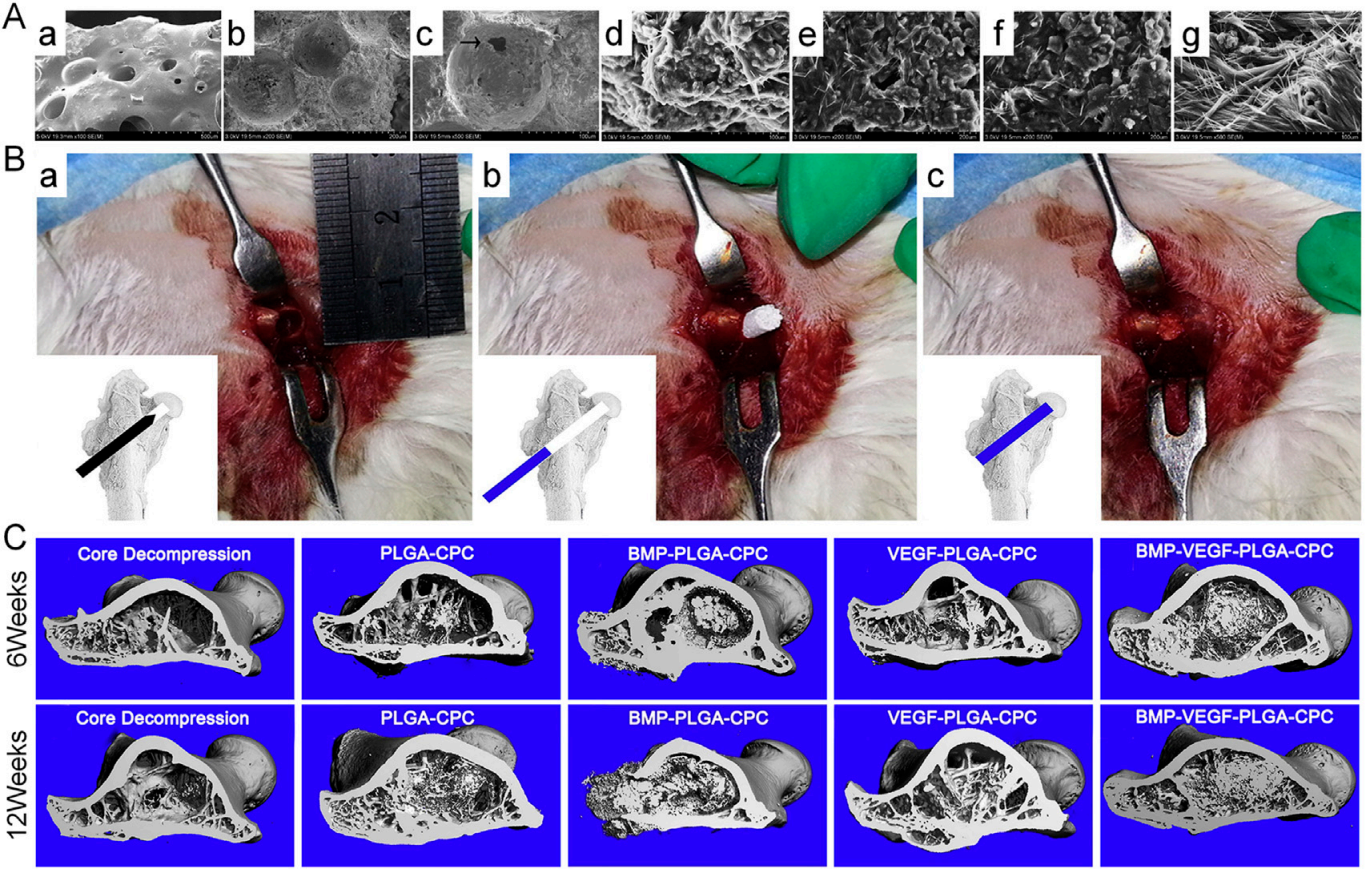
**(A)**: SEM micrographs of the surface (a), interior (b), and morphological features and microstructure of the BMP-VEGF-PLGA-CPC scaffold (c). (c) Interconnected micropores located on the porous walls of macropores in the scaffold (denoted by the arrowhead). BMSCs on PLGA-CPC scaffolds (d), BMP-PLGA-CPC scaffolds (e), VEGF-PLGA-CPC scaffolds (f) and BMP-VEGF-PLGA-CPC scaffolds (g). **(B)**: Rabbits undergoing CD (a) followed by implantation of scaffolds into the bone defect (b and c). **(C)**: Micro-CT evaluation of 3D reconstruction after 6 and 12 weeks of surgery. Reprinted with permission from Zhang H. X. et al. (2016).

Many studies have demonstrated that the combined use of BMP and VEGF is superior to the use of either factor and plays a synergistic role in promoting bone regeneration and vascularization (Grellier et al., 2009; Bai et al., 2014). Compared with the use of only VEGF, the combined use of BMP and VEGF increases bone mineral density and significantly enhances new bone formation (Chen et al., 2020). Zhang H. X. et al. (2016) used bone tissue engineering to combine BMP and VEGF for the treatment of ONFH and synthesized a novel calcium phosphate (CPC) composite scaffold containing BMP–VEGF-loaded poly-lactic-co-glycolic acid (PLGA) microspheres (BMP-VEGF-PLGA-CPC). The combination of BMP and VEGF synergistically promoted the adhesion, proliferation, osteogenic and angiogenic capabilities of BMSCs. Furthermore, rabbits with ONFH in the BMP-VEGF-PLGA-CPC group exhibited active osteogenesis and angiogenesis and higher recovery in bone necrosis. However, the pure CD group exhibited poor osteogenic and angiogenic activity and few changes in bone necrosis. Therefore, the combined application of CD and growth factors can improve blood circulation to the femoral head and promote the formation of new bones, potentially restoring the load function and preventing the collapse of the femoral head (Figure 1).

#### 4.1.2 Hydrogels

Injectable hydrogels offer a potential strategy for controlling the dissemination of biological molecules *in vivo* (Branco and Schneider, 2009; Gelain et al., 2010). Certain hydrogels, based on their composition, allow for minimally invasive delivery *via* injection, quickly transition from a solution to a gel in response to stimuli in the local environment, and are biodegradable. Therefore, hydrogels are ideal drug delivery vehicles (Hatefi and Amsden, 2002). Although hydrogels have been used for drug delivery in many studies, the infusion of a hydrogel into the femoral head has been less studied. Phipps et al. (2016) evaluated the potential of a peptide-based, self-assembling hydrogel called RADA16 to transition from a solution to a gel after its infusion into the femoral head, thereby preventing backflow, and examined its potential use as a delivery vehicle for BMP-2. After infusion, RADA16 was spread throughout the trabecular network of the femoral head and formed a gel *in situ*, with a slight leakage of the hydrogel. The bioactivity of BMP-2 was similar in cells treated with fresh BMP-2 and those treated with RADA16 hydrogels, indicating that the use of RADA16 did not alter the bioactivity of delivered proteins under certain conditions. In addition, the proliferation of BMSCs was significantly higher on RADA16 hydrogels than on tissue culture plastic, indicating that RADA16 offered a suitable matrix for supporting cellular proliferation. This novel strategy may be beneficial for the treatment of ONFH.

FGF-2 is a pleiotropic regulator of the proliferation, migration, and differentiation of cells in bone tissues and the vasculature and has anabolic effects on angiogenesis and bone formation (Nugent and Iozzo, 2000). Studies have shown that FGF-2 in gelatin hydrogels can be released consistently at relatively low concentrations (Nguyen et al., 2015). FGF-2 delivered *via* gelatin hydrogels has therapeutic potential in ischemic limb and heart injury and bone formation for fracture repair (Nakajima et al., 2004; Zhou et al., 2021). Therefore, FGF-2 encapsulated within gelatin hydrogels offers a promising strategy for the treatment of ONFH. Kuroda et al. (2010) investigated the potential effects of recombinant human FGF-2 (rhFGF-2) on bone repair in a rabbit model of ONFH. The treatment group was administered a single local injection of 100-μg rhFGF-2 in 100-μl gelatin hydrogel microspheres into the femoral head. The control group was administered phosphate-buffered saline in 100-μl gelatin hydrogel microspheres. Morphological, histopathological, and radiologic analyses showed the collapse of the femoral head and progression of articular cartilage degeneration in the control group 16 weeks after the injection. However, rhFGF-2 treatment resulted in new bone formation in the femoral head and prevented the femoral head from collapsing.

**FIGURE 2:**
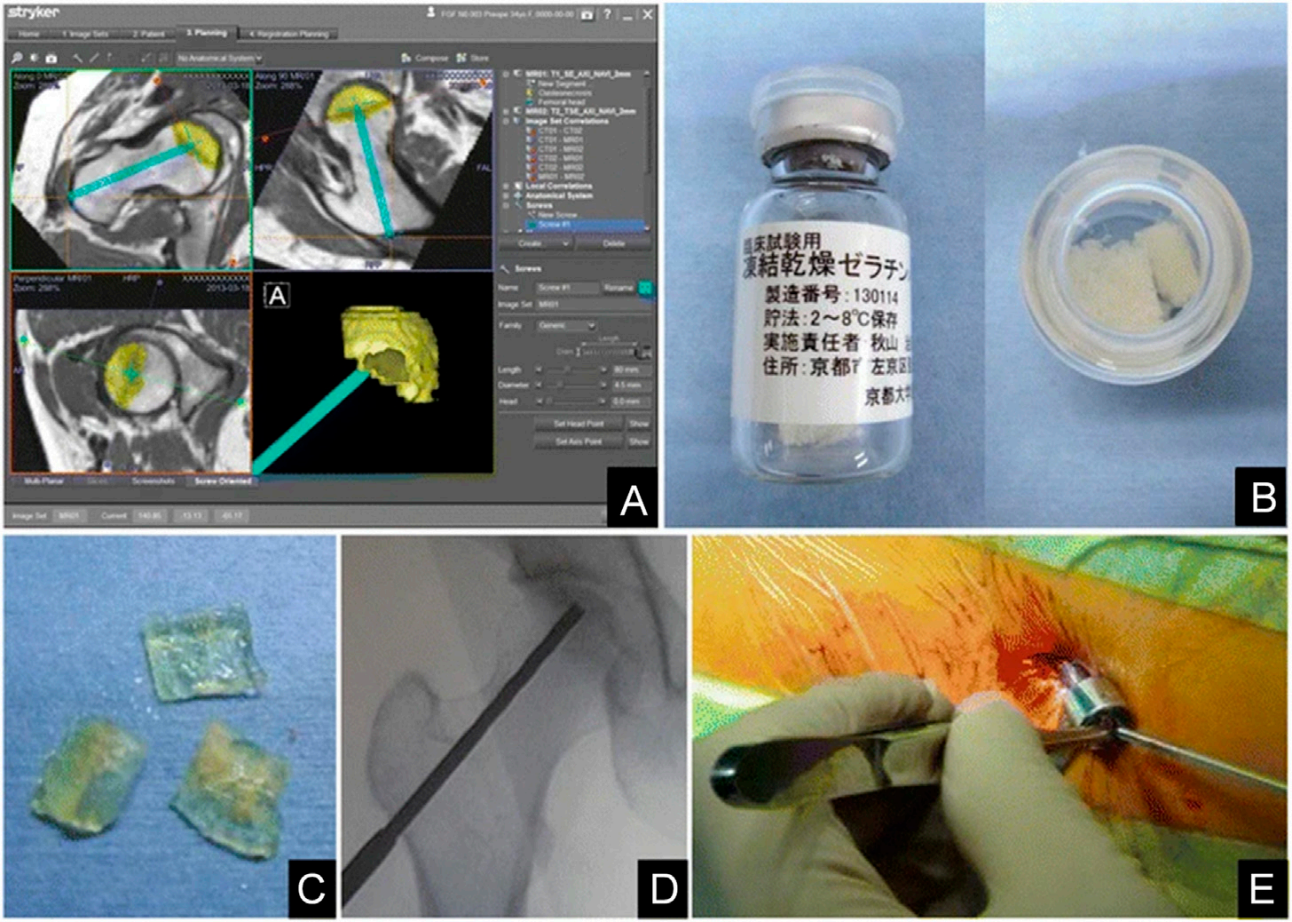
Controlled release of rhFGF-2 for the treatment of ONFH. **(A)**: Pre-operative planning. **(B)**: Preparation of rhFGF-2-encapsulated gelatin hydrogel. **(C)**: Pieces of the rhFGF-2-encapsulated gelatin hydrogel. **(D)**: Intra-operative fluoroscopic imaging after drilling. **(E)**: Percutaneous administration of the gelatin hydrogel. Reprinted with permission from Kuroda et al. (2016).

Furthermore, Kuroda et al. (2016) evaluated the safety and clinical outcomes of a single local injection of gelatin hydrogels impregnated with rhFGF-2 for treating the pre-collapse stage of ONFH. Patients with ONFH were administered a single local injection of 800-μg rhFGF-2 encapsulated within gelatin hydrogels and were followed up for 1 year. During the follow-up period, there were no complications related to either surgery or treatment with hydrogels, and all patients recovered without problems. Moreover, all clinical scores related to pain, daily activity rating, and hip joint functions significantly improved postoperatively. Therefore, local administration of rhFGF-2 was safe. This method offers the crucial advantage of being a minimally invasive percutaneous technique that facilitates early return to society and promotes bone regeneration in necrotic areas (Figure 2). Therefore, with further development, it can become a valuable treatment strategy for the pre-collapse stage of ONFH because it can be performed any time before the femoral head collapse, regardless of the cause.

### 4.2 Genetic engineering

During bone repair, the body is often unable to meet the requirements of growth factors owing to its limited ability to synthesize and secrete. Moreover, exogenous growth factor implantation is ineffective and works for a limited period. Therefore, some scholars have used gene therapy as a tool to deliver growth factors to treat osteonecrosis. With gene modification, genes related to osteogenic induction factors can be introduced to target cells, and the genetically modified cells can continuously express growth factors. This method allows the release of endogenous growth factors continuously and stably and targets the cells as required to promote osteogenesis and maintain the phenotype of osteoblasts. The combination of different growth factors and genetic engineering strategies for the treatment of ONFH has been summarized in Table 2.

#### 4.2.1 Bone morphogenetic protein

Bone regeneration can be achieved using various bioactive molecules with different efficiency. BMPs are a type of critical bioactive molecules involved in bone regeneration. They can initiate the complete bone formation cascade, including the migration and differentiation of MSCs (Wozney, 2002). BMP-2 exhibits excellent osteogenic potential and has shown efficacy in a series of clinical trials on spinal surgery and trauma orthopedic treatment (Rivera et al., 2013). However, a high dose of BMP-2 is required for systemic administration, which results in adverse complications such as ectopic bone formation and inflammatory responses (Shields et al., 2006).

In addition to bioactive molecules, BMSCs have been the subject of extensive research related to bone regeneration. BMSCs are characterized by their self-renewal and differentiation capability and can differentiate into osteoblasts, adipocytes, endothelial cells, and chondrocytes (Yim et al., 2014; Ruiz et al., 2016). Therefore, BMSCs are involved in normal bone metabolism and are considered ideal seed cells for cell therapy of various human orthopedic diseases (Liu et al., 2014). BMP-transfected MSCs possess better osteogenic potential than primary MSCs. The expression of BMPs and osteogenic and vascular trophic factors is significantly upregulated in BMP-modified MSCs (Hsieh et al., 2018; Kim et al., 2018).

Tang et al. (2007) investigated the effectiveness of adenovirus-BMP-2-transduced BMSCs for the treatment of ONFH. They induced ONFH *via* liquid nitrogen in goats and implanted the β-TCP scaffolds/gene-modified BMSCs in goats after CD. After 16 weeks of implantation, there was a femoral head collapse in the untreated group but not in the treated group, in which the femoral heads had an average density and intact surface. In addition, new bone, fibrous tissue, and lamellar bone were formed in the macropores of the scaffold, and the regenerated bone tissue had better mechanical properties. Katiella et al. (2016) reported that implantation of the BMSC–BMP-2 composite on magnesium alloy rods into rabbits prevented experimentally induced ONFH. After 12 weeks of implantation, all rabbits in the experimental group resumed normal activities without apparent visceral injury. In addition, macroscopic observations revealed that the femoral head of rabbits in the experimental group maintained normal contour and shape, and histological analysis revealed that the treatment group had better-arranged trabecular structures, inconspicuous boundary of the implantation area, and near-normal cancellous bone at the implantation site. Therefore, BMSCs transfected with BMPs can improve bone repair in ONFH.

#### 4.2.2 Vascular endothelial growth factor

Owing to its potent angiogenesis, VEGF has been widely and intensively investigated to promote vascularization in bone tissue engineering. Studies have shown that VEGF participates in the initial phase of ONFH (Varoga et al., 2009). Therefore, strategies for promoting vascular reconstruction should be developed to improve bone repair in ONFH.

VEGF-165 is the primary secreted form of VEGF in humans and the central effector molecule that exhibits strong potential for the proliferation and angiogenesis of undifferentiated endothelial cells *in vitro* (Solovyeva et al., 2020). Hang et al. (2012) established a canine model of ONFH through femoral neck osteotomy and subsequent repinning. After CD, BMSCs transfected with VEGF-165 were implanted into the canine models. After 12 weeks of implantation, histological analysis revealed that the periosteum of the femoral head in the transgenic group was smooth, the chondrocytes were arranged in order, the bone trabeculae were complete and arranged in order, and the osteoblasts within trabeculae were visible. In addition, immunofluorescence staining for von Willebrand factor revealed that the transgenic group had the most pronounced neovascularization in the necrotic area above the plane of osteotomy, with a significant increase in vascular density. However, the number of blood vessels did not increase in the necrotic area after implantation of non-transgenic BMSCs.

Previous studies have demonstrated that GCs downregulate VEGF expression in primary osteoblasts, and the loss of VEGF may contribute to the initial stage of GIONFH (Varoga et al., 2009). Therefore, VEGF plays a critical role in ONFH treatment and can be used as a therapeutic target.

#### 4.2.3 Hepatocyte growth factor

HGF is secreted by MSCs and acts as a multi-functional cytokine. It stimulates mitogenesis, cell motility, and matrix invasion, thus playing a central role in angiogenesis, tumorigenesis, and tissue regeneration (Makarevich et al., 2012). It suppresses cell apoptosis in an oxygen-poor environment (Vogel et al., 2010) and induces the osteogenic differentiation of BMSCs (Wen et al., 2018). HGF exerts its effects by binding to its receptor c-Met. HGF at different concentrations has different mechanisms to regulate the proliferation and osteogenic differentiation of MSCs. At low concentrations (20 ng/ml), HGF preferentially promotes the osteogenic differentiation of MSCs by increasing the expression and phosphorylation of c-Met and activating the Akt pathway. However, at high concentrations (100 ng/ml), HGF strongly induces proliferation by activating the ERK1/2 signaling pathway (Wen et al., 2012b). Wen et al. (2008) reported that *in vitro* transfection of BMSCs with replication-deficient recombinant adenoviral vectors expressing the human HGF gene (Ad-HGF) increased the concentration of HGF to 133 ng/ml after 1 week of transfection, which decreased to 19 ng/ml after approximately 2 weeks. This change in concentration rapidly increased HGF concentration after injury, which promoted the proliferation of BMSCs to produce the appropriate number of cells required for tissue regeneration. The subsequent decrease in HGF concentration promoted the differentiation of BMSCs for tissue repair. In addition, hormone-induced ONFH and traumatic ONFH models were constructed to validate that HGF-transfected BMSCs can repair bone tissue in ONFH *in vivo* (Wen et al., 2012a).

HGF can induce the secretion of VEGF by activating its receptor c-Met, followed by activation of the ERK1/2 and Akt signaling pathways (Van Belle et al., 1998; Wojta et al., 1999; Dong et al., 2001; Sengupta et al., 2003). In addition, it can promote angiogenesis to repair bone tissue in ONFH.

#### 4.2.4 Fibroblast growth factor

FGF plays a pivotal role in bone homeostasis (Novais et al., 2021). Disruption of the FGF gene dramatically decreases bone formation and bone mass in mice (Montero et al., 2000). In addition, FGF performs many desirable functions, including upregulation of VEGF in osteoblasts and activation of the proliferation, migration, and osteogenic differentiation of MSCs (Saadeh et al., 2000; Xiao et al., 2010; D’Mello et al., 2015).

The imbalance between bone regeneration by osteoblasts and bone resorption by osteoclasts contributes to the occurrence and development of ONFH (Tan et al., 2012). OPG and receptor activator of NF-κB ligand (RANKL) are critical factors for osteoclast differentiation, and bone resorption can directly affect bone cell function. OPG reduces the production of osteoclasts by binding to RANKL (Walsh and Choi, 2014). Furthermore, tumor necrosis factor-alpha (TNF-α) is a significant cytokine regulating bone homeostasis. On the one hand, it causes osteoclastogenesis by activating osteoclasts. On the other hand, it inhibits osteogenic differentiation, thereby destroying bone tissue (Osta et al., 2014). Peng et al. (2018) and Peng et al. (2019) combined FGF-transfected BMSCs with xenogeneic antigens of cancellous bone (XACB) to generate tissue-engineered bone (XACB/FGF/BMSCs) and implanted these BMSCs into the necrotic region of rabbits with early ONFH after CD. After 3, 6, and 12 weeks of implantation, the expression of OPG and RANKL was significantly increased in the femoral head. However, the expression of TNF-α remained low for up to 12 weeks after transplantation. Histological analysis showed that numerous new bone trabeculae were found, which were not clear with normal bone boundaries, and the repaired bone was similar to the normal cancellous bone.

Zhang et al. (2018) combined FGF-overexpressing BMSCs with XACB to construct tissue-engineered bone, which effectively promoted vascular regeneration and improved bone repair in ONFH. Therefore, FGF-transfected BMSCs are promising gene therapy tools for bone repair in ONFH.

#### 4.2.5 Platelet-derived growth factor-BB

PDGF is a glycoprotein with five dimeric isoforms: PDGF-AA, PDGF-BB, PDGF-AB, PDGF-CC, and PDGF-DD (Zhang Z. et al., 2021). Among the five isoforms, PDGF-BB is especially pleiotropic (Wang et al., 2018). Secretion of PDGF-BB by preosteoclasts increases the migration of MSCs and endothelial progenitor cells through the PI3K/Akt/FAK pathway and the differentiation of osteoblasts through the Sphk1/S1P pathway (Xie et al., 2014). PDGF-B is a ligand of platelet-derived growth factor receptor-beta (PDGFR-β), and their binding activates PDGF-BB/PDGFR-β signaling (Andrae et al., 2008), which is critical for vasculogenesis or angiogenesis (Su et al., 2020). Studies have shown that modifying circulating PDGF-BB levels can benefit patients with osteoporosis and other age-related diseases (Zaidi et al., 2021). The lack of PDGF-BB secretion by preosteoclasts is associated with bisphosphonate-related osteonecrosis of the jaw (BRONJ) in rats treated with zoledronate, which can be reversed with local PDGF-BB supplementation (Gao et al., 2021). In addition, PDGF-BB has beneficial immunomodulatory effects (Zhang J. M. et al., 2016). Owing to the pleiotropy of PDGF-BB, some scholars have used it for the treatment of ONFH (Tsubosaka et al., 2021). Guzman et al. (2021) transduced MSCs with a lentiviral vector carrying the human PDGF-BB gene under the control of the phosphoglycerate (PGK) promoter and assessed the proliferative rate, PDGF-BB expression, and osteogenic differentiation capacity *in vitro*. In addition, they evaluated the therapeutic effects of the transduced MSCs by injecting them into the bone tunnel during CD in an *in vivo* rabbit model of steroid-associated ONFH. The results showed that PDGF-BB-overexpressing MSCs accelerated cellular proliferation and osteogenic differentiation *in vitro*. Additionally, augmentation of CD with PGK-PDGF-BB-MSCs increased bone mineral density, osteoclastogenesis, and angiogenesis in the rabbit model. Therefore, PGK-PDGF-BB-MSCs as an adjunct to CD may reduce the progression of osteonecrosis and enhance bone regeneration and angiogenesis in the treatment of early-stage ONFH.

#### 4.2.6 Gene co-modification

During bone tissue repair in ONFH, the formation of new bone and interconnected blood vessels should be promoted, which enables nutrient transfer, oxygen exchange, waste removal, and the regulation of cellular signaling (Wan et al., 2020). Consequently, in addition to BMSCs transfected with a single gene, functional co-modification of BMSCs with synergistic genes may be more beneficial for the treatment of ONFH.

Ma et al. (2015) observed the therapeutic effects of BMSCs modified with VEGF-165 and BMP-2. They combined arthroscopic CD and transplantation of the modified BMSCs into the femoral head. After 8 weeks of surgery, the bone defect in the femoral head was repaired, bone quality was improved, and the duration of bone repair was shortened. This combination method improved the high intraosseous pressure and the pathological state of bone microcirculation obstacles. In addition, it provided seed cells for reconstruction of the femoral head to promote bone repair in ONFH. Peng and Wang (2017) combined BMP-2- and FGF-transfected BMSCs with a demineralized bone matrix (DBM) to repair bone tissue in a canine model of ONFH. After 12 weeks of implantation of DBM seeded with the engineered BMSCs into the necrotic femoral head, the newly generated bone area, neovascularization density, and compression and bending strength parameters in the treated group were superior to those in the control group.

**FIGURE 3:**
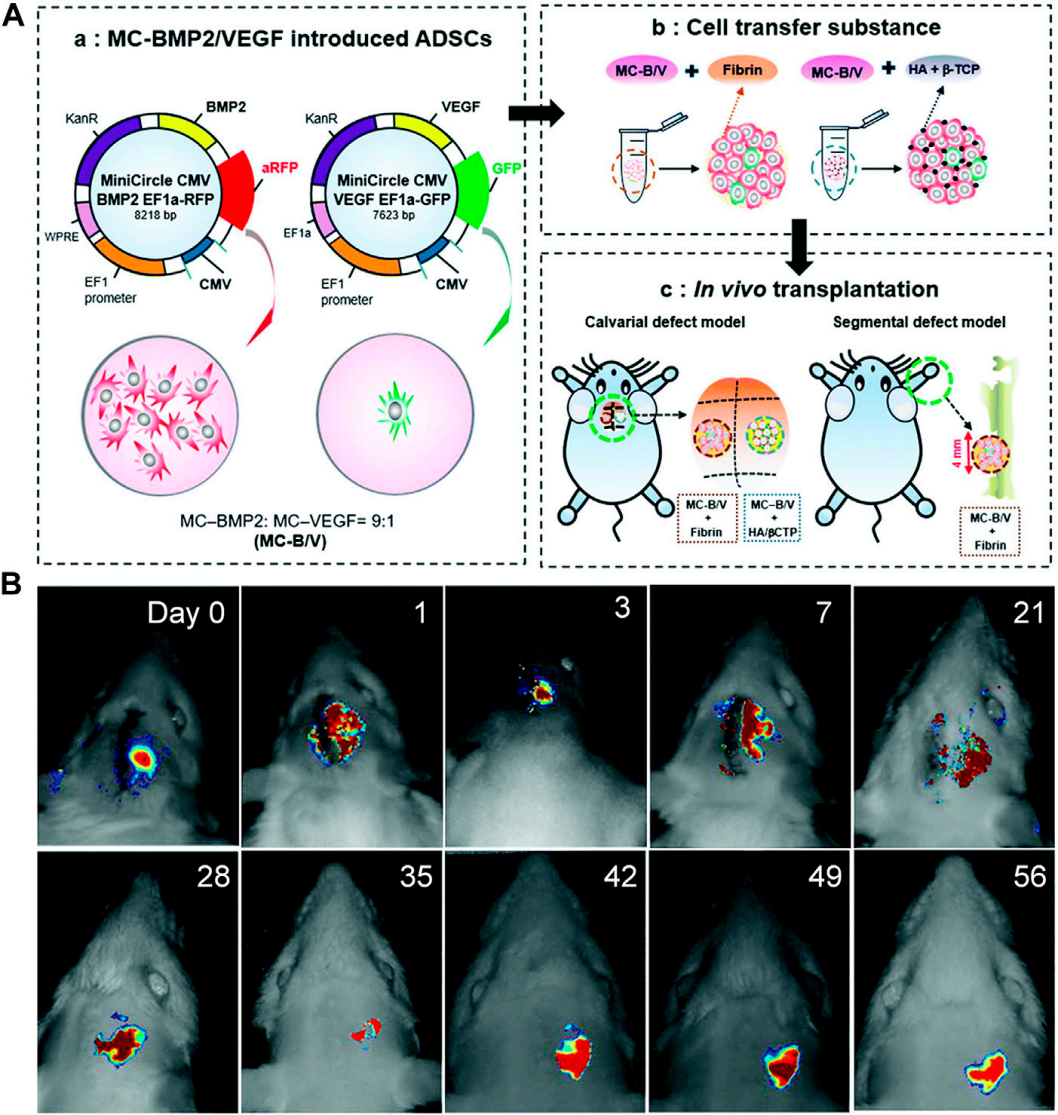
**(A)**: Development of BMP2/VEGF-transfected hASCs. **(B)**: *In vivo* tracking of implanted hASCs. Reprinted with permission from Lee et al. (2019).

However, the effectiveness of the combined gene transfer and the optimal combination ratio of two or more genes remain unknown. This information is essential because an excess of either factor may lead to undesirable and, occasionally, reverse effects (Itoh et al., 2001; Niida et al., 2005). Lee et al. (2019) transfected adipose stem cells (ASCs) with BMP2/VEGF to promote osteogenesis and angiogenesis simultaneously. The optimal ratio of BMP2-to-VEGF was determined to be 9:1. BMP2/VEGF-transfected ASCs administered in this ratio effectively healed critical-size calvarial defects and long-bone segmental defects in immunosuppressed rats (Figure 3). These findings provide a theoretical and experimental basis for the use of combined gene transfer in the treatment of ONFH or other orthopedic diseases.

### 4.3 Other strategies

#### 4.3.1 Combined with implanted bone

Although several procedures are used to preserve the femoral head in patients with ONFH in clinical settings, there is no consensus regarding the optimal procedure. In 1994, Rosenwasser et al. (1994) proposed the concept of the “light bulb procedure,” in which necrotic lesions were replaced with bone grafts *via* the window on the femoral head-neck junction without any damage to the joint cartilage. This procedure provides strong structural support for the femoral head, amends the morphology of the femoral head to a certain extent, and prevents further collapse of the femoral head. In addition, it has several advantages, including the simple surgical technique, low complication incidence, and straightforward surgical duration (Sun et al., 2014).

**FIGURE 4:**
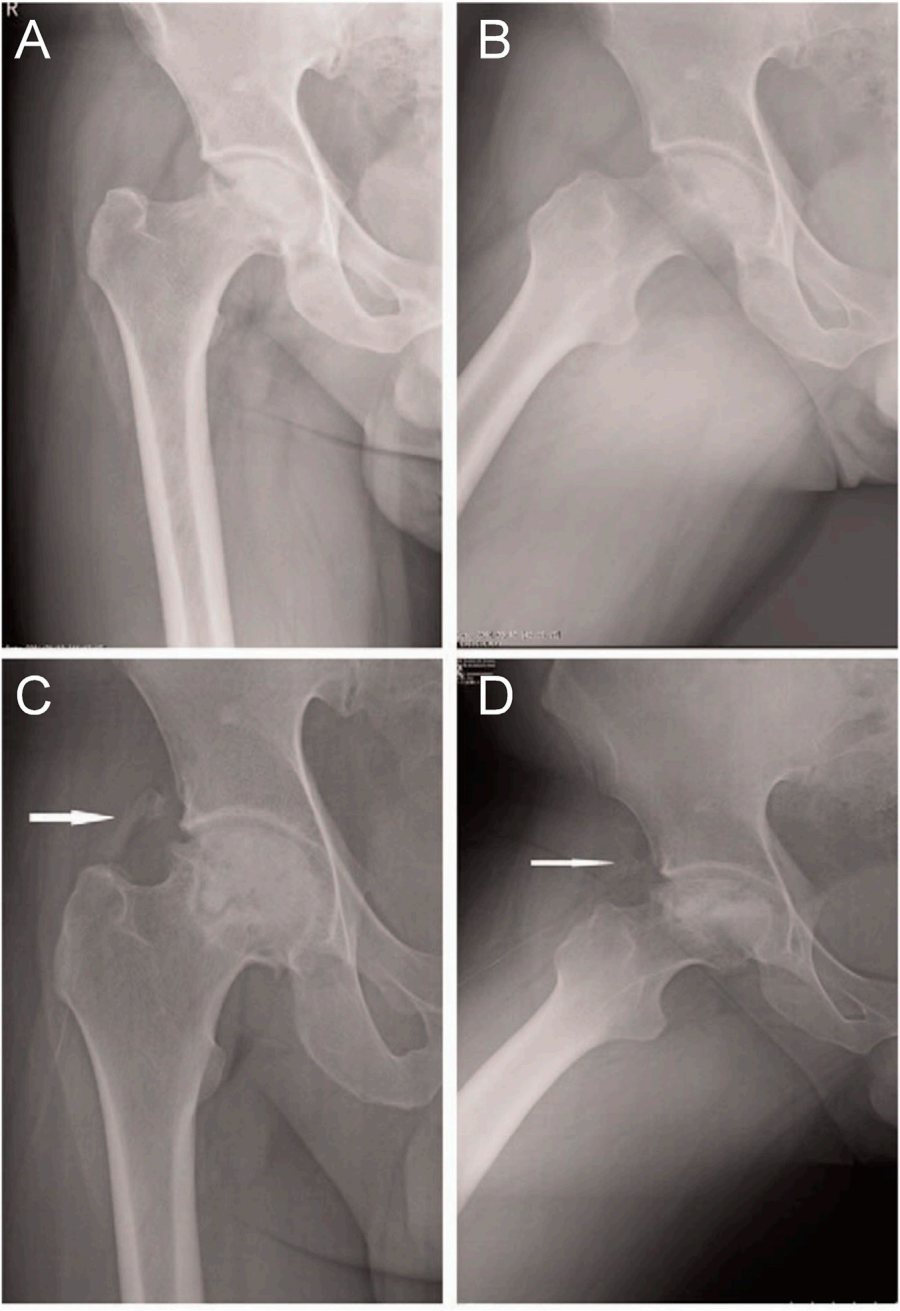
**(A)**: Osteonecrosis of the right femoral head in a 36-year-old male patient with ARCO stage IIIa disease. Graphs **(A)** and **(B)** represent the preoperative anteroposterior and frog-leg lateral X-ray images of the right hip, respectively. Graphs **(C)** and **(D)** represent the postoperative anteroposterior and frog-leg lateral X-ray images, respectively, after 4 months of surgery, showing a lamellar fragment of HO (class II) in the anterolateral soft tissues of the hip joint (white arrow). ARCO, Association Research Circulation Osseous; HO, heterotopic ossification. Reprinted with permission from Shi et al. (2017).

In a retrospective study, Sun et al. (2014) analyzed the clinical efficacy of rhBMP-2 in the treatment of ONFH. A total of 46 patients with nontraumatic ONFH (79 hip specimens) were included and divided into two groups. The first group of patients was subjected to the light bulb procedure and rhBMP-2 treatment, whereas the second group was subjected to the light bulb procedure alone. After follow-up, the results showed no significant differences in clinical results between the two groups. However, the combination method may effectively prevent the requirement for hip replacement in younger patients with early-stage ONFH. Although no significant difference was observed in clinical results, radiological analysis revealed that rhBMP-2 improved the speed and quality of bone repair inside lesions. Shi et al. (2017) conducted the same retrospective analysis involving 94 patients with non-traumatic ONFH (141 hip specimens), and the experimental results were consistent with those of previous studies. Although patients in the experimental group had a high rate of femoral head preservation and high HHS scores, statistical analysis showed no significant differences (Figure 4).

#### 4.3.2 Local injection

In addition to debridement of the necrotic femoral head and implantation of new bone, other treatment options involve direct injection of drugs into the femoral head, which can avoid additional surgical intervention (Gao et al., 2013). However, the traditional administration of growth factors is limited by their relatively short half-lives and potential side effects (Aldridge et al., 2005). Therefore, controlled and more continuous administration may be required to ensure the effective activity of growth factors (Li et al., 2021). Dailiana et al. (2018) injected VEGF into the femoral head of a canine model of ONFH using an osmotic micropump. After 12 weeks of injection, histological analysis showed that local treatment with VEGF led to bone tissue remodeling and new bone formation. VEGF acted in a dose-dependent manner, indicating that the optimal concentration of VEGF is critical for therapeutic outcomes and may depend on the size of the necrotic area. Therefore, the delivery model of VEGF should be further optimized to achieve better bone regeneration.

The imbalance between osteogenesis and osteolysis plays an essential role in the occurrence and development of ONFH. Bisphosphonates are potent inhibitors of osteoclast-mediated bone resorption, which inhibit osteoclasts and reduce femoral head deformities (Ma et al., 2021). Local administration of zoledronic acid in the femoral head has positive effects on the bone structure of the femoral head in modified rat models of traumatic ONFH (Zhao J. et al., 2021). Although monotherapy with bisphosphonates can maintain the structural integrity of the necrotic epiphysis, the lack of new bone formation has been observed in studies on large animal models (Kim et al., 2005). Vandermeer et al. (2011) injected ibandronate (IB) combined with BMP-2 into immature pig models of ONFH *via* percutaneous intraosseous injection. After 8 weeks, the pigs were sacrificed, and further analysis showed that simultaneous local administration of ibandronate and BMP-2 improved preservation of the spherical shape of the femoral head and stimulated bone healing in immature pig models of ischemic osteonecrosis.

Furthermore, Aruwajoye et al. (2017) investigated the effects of IB and BMP-2 on the mineral content and nanoindentation properties of the bone after ONFH. Backscattered electron imaging, Raman spectroscopy, and nanoindentation testing showed that compared with IB monotherapy, treatment with BMP-2 or IB/BMP-2 improved restoration of the average mineral content and nanomechanical properties after ONFH. Therefore, simultaneously inhibiting osteolysis and promoting osteogenesis can improve bone repair in ONFH.

#### 4.3.3 Combined with low-intensity pulsed ultrasonography

Low-intensity pulsed ultrasonography (LIPUS) can enhance osteogenic differentiation of MSCs, stimulate the differentiation and proliferation of osteoblasts, inhibit osteoclasts, improve local blood perfusion and angiogenesis, and accelerate the healing of stress fractures (Chan et al., 2006; Amini et al., 2020). Clinical studies have shown significant positive effects of LIPUS in treating fresh (Shimizu et al., 2021) and nonunion (Jiang et al., 2019; Leighton et al., 2021) fractures. In addition, LIPUS is considered a non-invasive treatment strategy for ONFH (Yan et al., 2011). However, other effective strategies for minimizing the treatment duration and improving patient outcomes should be investigated intensively.

Zhu et al. (2020) combined BMP-2 and LIPUS for treating ONFH. They prepared a novel nanofiber scaffold with sustained release of BMP-2 and implanted the scaffold into the femoral neck of rats. Subsequently, LIPUS was used to observe the effects of the scaffold on bone repair in murine models of ONFH. Compared with the use of LIPUS alone, the sustained release of BMP-2 from nano-scaffolds enhanced bone regeneration, resulting in superior bone quality. In addition, treatment with the scaffolds increased the number and diameter of blood vessels and promoted angiogenesis. Therefore, the combined application of LIPUS and BMP-2 contributes to bone formation and repair in ONFH.

## 5 Current limitations and future prospects

ONFH is a progressive disease with complex etiology and unclear pathogenesis and lacks optimal treatment, especially for young patients. Multiple growth factor-related genes play a crucial pathogenic role in ONFH, especially those involved in osteogenesis and angiogenesis. In addition, abnormalities in growth factor-related signaling pathways are also involved in the pathological process of ONFH. Growth factor-based therapy for ONFH has developed from relevant basic research to clinical application and have been demonstrated to be effective. Growth factors can stimulate both angiogenesis and osteoinductive stem cell differentiation. Additionally, they can enhance cell proliferation and bone regeneration, thereby assisting in bone repair in osteonecrosis. They play an essential role in osteonecrosis because any therapy that can accelerate osteogenesis or angiogenesis can potentially improve therapeutic outcomes and quality of life. The advantage of using growth factors to treat ONFH is the avoidance of additional surgical interventions because most growth factors can be not only injected but also used in combination with surgical treatments and tissue engineering materials.

However, the relatively short half-life of growth factors under physiological conditions makes it difficult to achieve sustained and controlled delivery. Genetic engineering, involving precise manipulation of cellular DNA sequences to alter cell fates and organism traits, offers the potential to both understand human genetics and cure genetic disease. Therefore, transfection of growth factor-related genes into cells *via* a carrier can enhance the regenerative capacity of the cells by initiating a heightened signaling response for cellular recruitment and initiation of anabolic activity. In addition, it can increase the autocrine activity of cells to enhance intrinsic mechanisms of bone repair, making it a powerful gene therapy tool for the treatment of ONFH.

Traditional surgical approaches, such as bone grafts, vascular implants, and metal implants, have been used in combination; however, these treatments are partially successful because they neither initiate the regeneration of bone tissue nor promote angiogenesis. Although the implant provides good mechanical support, it has absolutely no effect on bone regeneration. The development of bone tissue engineering offers an excellent solution for the treatment of ONFH. The bone tissue engineering scaffold has a strong biological function and provides mechanical support for the femoral head, thus preventing collapse of the weight-bearing area of the femoral head. Importantly, with the development of various growth factor-based delivery systems, growth factors can be loaded onto the surface of bone tissue engineering scaffolds *via* physical, chemical, and biological binding. Such scaffolds allow for a continuous slow release of growth factors at the necrotic site and stimulate osteoblast growth and induction of bone formation during bone regeneration, thereby supporting vasculature at the necrotic site. In addition to bone tissue engineering scaffolds, hydrogels have exhibited good biocompatibility, degradability, and injectability. Hydrogels act as carriers and can be incorporated with growth factors. Owing to the degradation of hydrogels and the diffusion movement of growth factors after injection into the site of osteonecrosis, slow and controlled local release can be achieved for treating ONFH.

However, several issues and challenges should be addressed before the practical application of growth factors in the treatment of ONFH. Some adverse effects may result from the uncontrolled expression of transgenes. For instance, overexpression of HGF may lead to the development of sarcoma and negatively affect osteogenesis and bone formation; uncontrolled synthesis of VEGF may induce angiomas and, in turn, impair osteogenesis; overexpression of BMP can lead to ectopic ossification and even tumorigenesis. Therefore, more precise and controllable gene transfer strategies should be developed in the future. The choice of vectors for gene delivery is an important influencing factor in genetic engineering. Viral vectors enable long-term and consistent transgene expression by integrating the transgene into the host genome but impose a risk of insertional mutations and tumor formation. Although non-viral vectors have great potential for gene delivery, especially owing to their safety, their low delivery efficiency remains the major problem. Therefore, it is necessary to develop safer and more effective techniques for genetic manipulation.

rhBMP-2 and rhBMP-7 have been approved by the Food and Drug Administration (FDA) for clinical treatment of vertebral fusion, open or nonunion fractures, and maxillofacial bone reinforcement. When growth factors are used as monotherapy or are combined with other strategies for the treatment of ONFH, owing to the short half-life and potential side effects of growth factors, the amount, use mode, release mode, and release time should be accurately optimized to achieve appropriate, safe, effective, and long-term effects. Bone tissue engineering materials used in combination with growth factors should have excellent mechanical properties and should be able to withstand dynamic, physiological compressive and shear loads at the site of implantation to support the necrotic site until new bone is formed. In the present study, a combination of natural and synthetic materials and porous metal scaffolds exhibited promising mechanical properties for bone defect repair. Therefore, research on composite materials incorporated with growth factors may help to develop more effective tissue engineering-based strategies for the treatment of ONFH.

## 6 Conclusion

Polymorphisms in osteogenic and angiogenic growth factor-related genes and abnormalities in their associated signaling pathways are involved in the development and progression of ONFH, which offers novel insights into the prevention and treatment of ONFH. Given the important regulatory role of growth factors in bone regeneration, they can be used for genetic and tissue engineering to develop novel strategies for the treatment of ONFH. However, developing such strategies is challenging owing to the unknown safety and efficacy of growth factors. Therefore, further basic and translational studies are required to maximize the clinical potential of growth factor-based diagnostics and therapeutics for ONFH.

## Data Availability

The original contributions presented in the study are included in the article/Supplementary Material, further inquiries can be directed to the corresponding author.
